# Immunoinformatic based identification of cytotoxic T lymphocyte epitopes from the Indian isolate of SARS-CoV-2

**DOI:** 10.1038/s41598-021-83949-9

**Published:** 2021-02-25

**Authors:** Viswajit Mulpuru, Nidhi Mishra

**Affiliations:** Department of Applied Science, Indian Institute of Information Technology Allahabad, Prayagraj, 211012 India

**Keywords:** Computational biology and bioinformatics, Immunology

## Abstract

The Severe acute respiratory syndrome coronavirus 2 (SARS-CoV-2) has turned into a pandemic with about thirty million confirmed cases worldwide as of September 2020. Being an airborne infection, it can be catastrophic to populous countries like India. This study sets to identify potential cytotoxic T lymphocyte (CTL) epitopes in the SARS-CoV-2 Indian isolate which can act as an effective vaccine epitope candidate for the majority of the Indian population. The immunogenicity and the foreignness of the epitopes towards the human body have to be studied to further confirm their candidacy. The top-scoring epitopes were subjected to molecular docking studies to study their interactions with the corresponding human leukocyte antigen (HLA) system. The CTL epitopes were observed to bind at the peptide-binding groove of the corresponding HLA system, indicating their potency as an epitope candidate. The candidacy was further analyzed using sequence conservation studies and molecular dynamics simulation. The identified epitopes can be subjected to further studies for the development of the SARS-CoV-2 vaccine.

## Introduction

Severe acute respiratory syndrome coronavirus 2 (SARS-CoV-2) is a virus of the genus *Betacoronavirus* and family *Coronaviridae* with symptoms ranging from a runny nose to severe acute respiratory syndrome and kidney failure^1^. It has infected over 150 countries worldwide and the World Health Organization has declared it a pandemic. An airborne infection like SARS-CoV-2 in populous countries such as India can escalate very rapidly. Thus, finding a vaccine candidate for this deadly disease is of priority to researchers worldwide.

It is known that cytotoxic T lymphocytes (CTL) with the help of specialized proteins known as T cell receptors (TCR) on their surface help fight virally-infected cells. Using this natural mechanism of the immune system to fight this dreadful virus can be of great importance in vaccine development. Each TCR that is present on the surface of the CTL can specifically identify antigenic peptides bound to the major histocompatibility complex (MHC). Whenever the TCR detects a viral antigen, the CTL releases cytotoxins to kill the virus-infected cell thus helping in immune response^2^.

Vaccine development is a tedious and complex process lasting for several years. With the advancement of bioinformatics, sequence‐based techniques have rapidly improved providing information related to the genome and proteome of different viruses. Employing these bioinformatics tools, scientists and researchers are greatly reducing the time and cost required for the experiments. In vaccine development, by analyzing the genomic and proteomic data, the conserved and immunogenic epitopes from a pathogen can be predicted effectively by sequence similarity studies. This helps in reducing the time required for epitope identification before preclinical and clinical trials. The computational techniques can also be employed to study the effects of the epitopes towards various cells and entities involved in the immune response by simulating an in-vivo environment thus increasing the efficiency of vaccine development.

This study aimed to identify cytotoxic T cell (CTL) epitopes of SARS-CoV-2 Indian isolate for designing potential vaccine candidates that are effective on the Indian population using an in-silico approach. We predicted the CTL epitopes for all human leukocyte antigen supertypes (HLA) which have a high allelic frequency in the Indian population. We further studied the immunogenicity, foreignness, and interactions between the epitopes and the HLA molecules by the means of molecular docking studies. The stability of the docking studies was confirmed by employing molecular dynamics simulation analysis.

## Materials and methods

### Retrieving the complete genome and the coding sequence from an Indian isolate

The amino acid sequence of the complete genome of Severe acute respiratory syndrome coronavirus 2 (SARS-CoV-2) isolate of India (MT050493.1) with 9950 AA was retrieved from NCBI. All the coding regions of the genome were retrieved from the features section containing the coding sequences of orf1ab polyprotein, surface glycoprotein, orf3a protein, envelope protein, membrane glycoprotein, orf6 protein, orf7a protein, orf8 protein, nucleocapsid phosphoprotein, and orf10 protein.

### Prediction of cytotoxic T cell epitopes for the Indian population

NetCTLpan version 1.1^3^ was used to predict the CTL epitopes across the proteins coded by the SARS-CoV-2 Indian isolate. NetCTLpan uses a neural network to predict TAP-transporter binding and C terminal cleavage predictions in addition to HLA binding prediction. Considering the HLA supertype variation across populations, we predicted the epitopes only for those HLA supertypes which constitute the majority of human leukocyte antigen (HLA) distribution in the Indian population keeping cutoffs and parameters of NetCTLpan as default. The study on the evolution of HLA-A and HLA-B polymorphisms reveals that HLA A3, B7, and B44 are the major HLAs present in the Indian population^4^ with HLA A3 constituting HLA-A type in 47 percent of the Indian population and HLA B7, B44 constituting HLA-B type in 30 percent and 28 percent of the Indian population respectively.

### Prediction of epitope immunogenicity

Although the binding affinities of the peptides towards HLA help in predicting the epitopes, the immunogenicity plays an important role in the immune response. All the predicted epitopes were subjected to the Immune Epitope Database (IEDB) immunogenicity tool^5,6^ to predict their immunogenicity score. IEDB immunogenicity tool relies on physicochemical properties such as side chain composition, amino acid position to predict the immunogenicity of the peptide sequence.

### Identification of unique epitopes

As the healthy human body majorly shows immune response only towards foreign antigens except under certain conditions such as autoimmune disorders, it is of great importance to consider only those epitopes which are foreign to the human body as a potential vaccine epitope candidate. To identify the vaccine epitope candidates that are foreign to the human body, all the epitopes that show positive immunogenicity are subjected to the Multiple Peptide Match tool^7^ against human reference proteome with Proteome ID UP000005640. The Peptide Match tool is a search engine based on Apache Lucene and is designed to quickly retrieve all occurrences of the given query peptides from a reference or specified proteome.

### Docking studies

The top two percent of the foreign epitopes based on immunogenicity scores were selected. To further confirm the candidacy of the foreign epitopes for vaccine development, these epitopes were subjected to molecular docking studies to confirm their interactions with the specified HLA at the peptide-binding groove considering PDB IDs 6O9C, 6AT5, and 3KPS for the structures of HLA-A3, HLA-B7, HLA-B44 respectively. This molecular docking study was performed using HPEPDOCK Server^8^. The docking was performed without specifying the binding site residues to investigate if the studied epitopes would bind at the peptide-binding groove without any lead. The interaction diagrams are generated using LigPlot+^9^.

### Conserved nature of the selected epitopes

To check the conserved nature of the selected epitopes from Indian isolates of the virus, The SARS-CoV-2 genomic sequences isolated in the Indian region were obtained from GISAID^10^ and aligned using the MAFFT sequence alignment server^11^. All the 2084 genomic sequences as of 15 September 2020 were downloaded from the EpiCoV repository of the GISAID. Later, the alignment was translated using the standard genetic code and the locations containing the epitopes were extracted from the alignment. The extracted alignment was subjected to WebLogo^12^ to generate sequence logos for visualizing their conserved nature in the Indian isolates.

### Molecular dynamics studies

To study the stability of the HLA-epitope interactions, the structures from the docking studies were subjected to all-atom molecular dynamics simulations to explore their stability and conformational flexibility using CHARMM36 all-atom force field^13^ and GROMACS (Version 2018.2)^14^. The complex was solvated using TIP3P explicit water molecules and in-house ad hoc scripts were used to neutralize, minimize, and equilibrate using GROMACS. The neutralization was performed using Cl− and Na+ ions as needed while the minimization was performed using the steepest descent algorithm until the maximum force is less than 10.0 kJ/mol. The system was equilibrated using NPT conserved ensemble under constant pressure and temperature of 1 Bar and 300 K respectively. Further, the molecular dynamics simulations of 50 ns were performed using the leapfrog algorithm with an integration time step of 2 fs. The generated trajectories were analyzed using GROMACS analysis utilities to derive results. The graphs showing the root mean square deviation (RSMD) between the initial and the simulated structure, the change in coulombic interaction energies between HLA and epitope over the simulation time, and the change in the number of hydrogen bonds formed between HLA and epitope are plotted to determine the stability of the HLA-epitope complex.

## Result and discussion

253 CTL epitopes are predicted by NetCTLpan across the proteins coded by SARS-CoV-2 Indian isolate towards HLA A3, B7, and B44. The epitopes as predicted by NetCTLpan are shown in Supplementary Table S1 online. The scores of the peptides constitute combined scores of HLA binding, TAP-transporter binding, and C terminal cleavage prediction scores.

While there are two classes of MHC molecules namely MHC I and MHC II, in this study only HLAs corresponding to MHC class I are considered due to their widely known functions of viral peptide presentation to CD8 + T cells during viral infections^15,16^. The MHC class I are also known to be ubiquitous while MHC class II molecules are known to be present only on select antigen-presenting cells^17^. There are also instances of viruses inhibiting MHC class II antigen presentation^18^.

The IEDB immunogenicity tool was used to calculate the immunogenicity of the epitopes as it plays an important role in examining the immune response. IEDB immunogenicity tool returned 139 epitopes with positive scores. The data showing the immunogenicity score of the epitopes are shown in Supplementary Table S2 online.

All the epitopes with positive immunogenicity scores are subjected to the Multiple Peptide Match tool to identify the epitopes that are foreign to the human body. It was observed that all the epitopes that showed positive scores for immunogenicity calculations were foreign to the human proteome. The peptide matching step was performed on the sequence dataset ‘UniProtKB release 2020_01 plus isoforms |SwissProt| Isoform’ with the target organism set as ‘Homo sapiens [9606]’. The output log had stated that 0 out of 139 unique peptides had matches in 0 protein(s) found in 0 organism(s) confirming their foreignness to the human body. Ultimately, to select the promising epitopes, the NetCTLpan score and immunogenicity score were employed. The NetCTLpan score was used to filter the highly likely CTL epitopes by eliminating the epitopes with a negative NetCTLpan score. The remaining epitopes were sorted based on their immunogenicity score to identify the top epitope candidates.

Although there were many peptide-based vaccines against various infections in different phases of development, none of these have either been concluded or had their sequence data directly available. So, we have considered an epitope from protein Superoxide Dismutase of *Mycobacterium tuberculosis* that showed promising results in human subjects as a control epitope in this study^19^. The control epitope and the top five vaccine epitope candidates amounting to the top two percent of the total 253 epitopes as predicted by the above steps along with their epitope prediction score by NetCTLpan and immunogenicity score are given in Table 1.

**Table 1:** The epitope prediction score and immunogenicity score of the top five vaccine epitope candidates.

Epitope	Protein	HLA	NetCTLpan score	Immunogenicity score
DMWEHAFYL	Control	HLA-A02	1.03873	0.33762
LVAEWFLAY	orf1ab polyprotein	HLA-A03	0.70209	0.45285
WPWYIWLGF	surface glycoprotein	HLA-B07	0.68398	0.41673
REHEHEIAW	orf1ab polyprotein	HLA-B44	0.82399	0.37218
HVTFFIYNK	orf3a protein	HLA-A03	0.78979	0.36278
SPRWYFYYL	nucleocapsid phosphoprotein	HLA-B07	1.00321	0.34101

**Figure 1:**
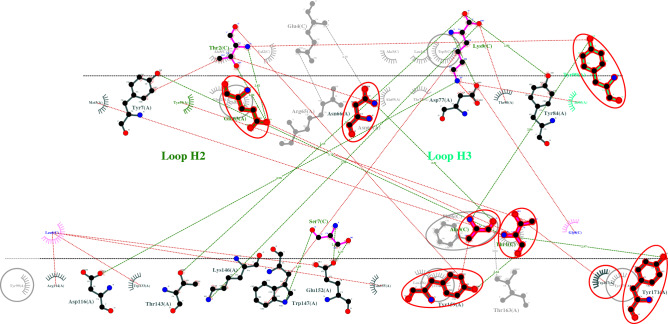
The interactions between HLA-A3 (PDB ID: 6O9C) and the epitope LVAEWFLAY from the SARS-CoV-2 proteome.

**Figure 2:**
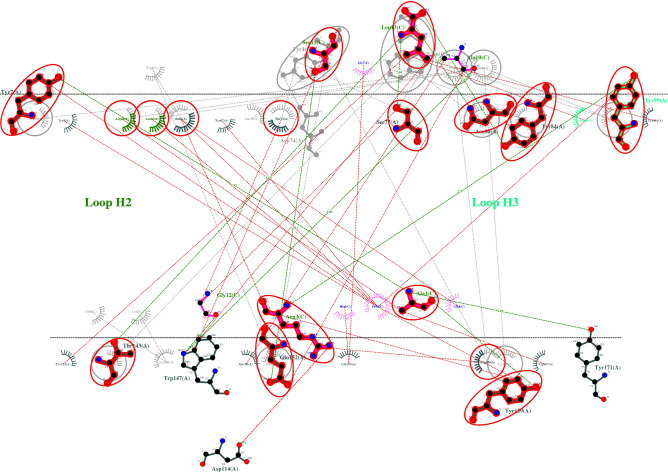
The interactions between HLA-B7 (PDB ID: 6AT5) and the epitope WPWYIWLGF from the SARS-CoV-2 proteome.

**Figure 3:**
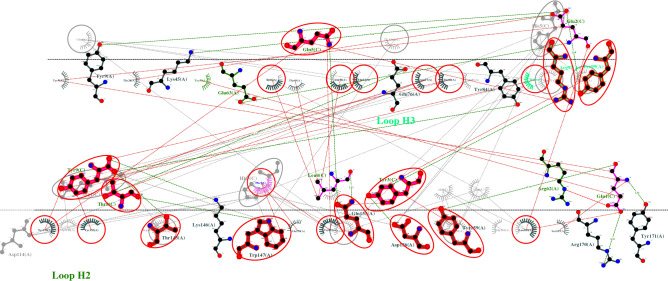
The interactions between HLA-B44 (PDB ID: 3KPS) and the epitope REHEHEIAW from the SARS-CoV-2 proteome.

**Figure 4:**
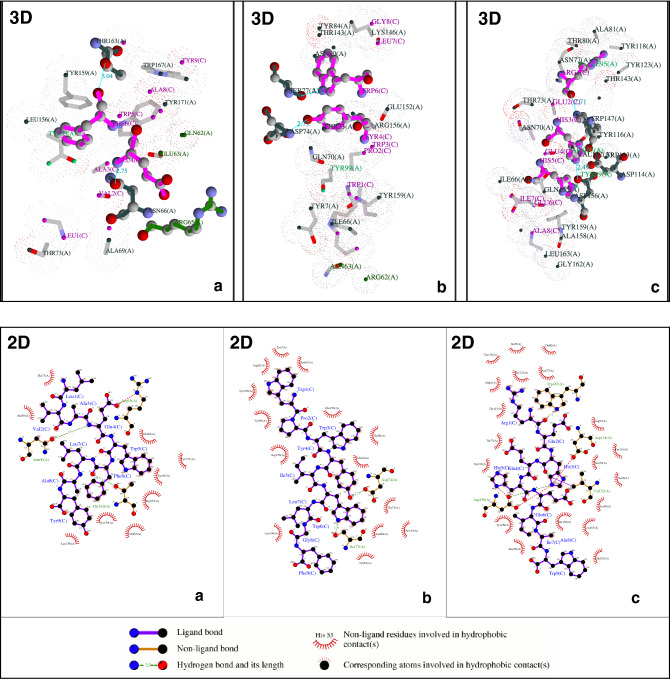
The two-dimensional and three-dimensional representation of the interactions between the identified epitope and the corresponding HLA. (**a**) HLA-A3—LVAEWFLAY. (**b**) HLA-B7—WPWYIWLGF. (**c**) HLA-B44—REHEHEIAW.

The top three vaccine epitope candidates based on their immunogenicity score are subjected to molecular docking studies using HPEPDOCK Server. Although the top two percent of the predicted epitopes were selected for docking studies, coincidentally the top three selected epitopes were against different HLAs covering all the HLAs from the study. So, the docking studies were performed only for the best epitope candidate for each HLA. The interaction diagrams revealed that the peptide epitopes bind to the peptide-binding groove even though they were subjected to blind docking. Thus, confirming their vaccine candidacy. To visualize the equivalent interactions between the reference and the epitope towards the corresponding HLA, the interaction plots of the docked epitopes and the references are shown in Figs. 1, 2, and 3 by superposing the reference peptide-HLA interaction plot (foreground) onto the epitope-HLA interaction plot (background) where the peptide molecule already bound to the HLA in the PDB structure file is considered as the reference. The equivalent interactions between the reference peptide available in the PDB file and the studied epitope are circled in red to facilitate identification. The hydrogen bonds are represented by a green dotted line along with their distance while the non-bonded interactions (salt bridges and hydrophobic interactions) are represented by a red dotted line. The residues from different loops of the heavy chain are represented by different shades of green. Chains A and B are the chains corresponding to the protein while the chain C is the epitope. The two-dimensional interaction diagrams tracing the whole backbone of the studied epitopes are shown in Fig. 4. The three-dimensional representation of the interactions between the identified epitope and the corresponding HLA is also shown in Fig. 4 with hydrogen bonds in cyan and nonbonded interactions in dot surfaces. The epitope residues are represented in magenta either by balls or intra-residue bonds.

**Figure 5:**
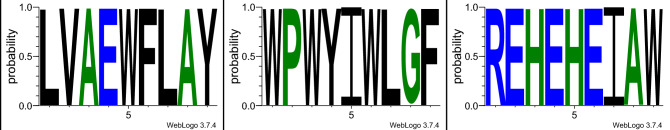
The sequence logos of the identified epitopes.

As it is observed that the Interactions between the identified epitopes are similar to that of the reference peptides, the conserved nature of the selected epitopes was studied and the sequence logos as generated by WebLogo is shown in Fig. 5 showing their probability of occurrence. From the sequence logos, we conclude that the identified epitopes are highly conserved in the Indian isolates of the virus.

**Figure 6:**
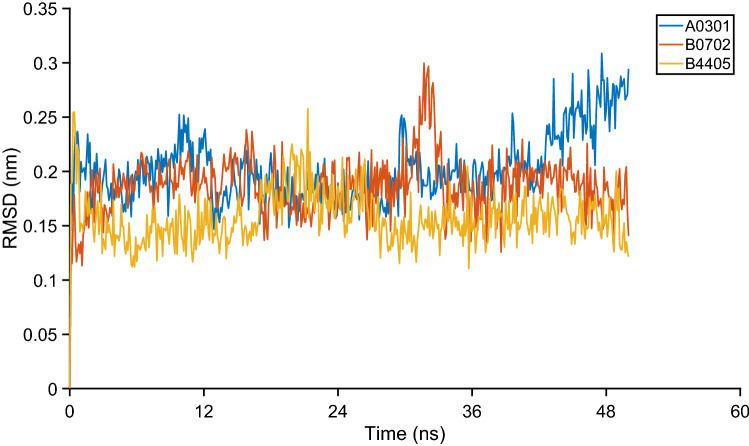
The RMSD plot of the HLA-epitope complex.

To check the HLA-epitope stability of the conserved epitopes, a molecular dynamics simulation of 50 ns was performed. The RMSD plot as shown in Fig. 6 suggests that the docked HLA-epitope complexes are highly stable with an average RMSD of 0.2 nm, 0.19 nm, and 0.16 nm for HLA-A*03-epitope, HLA-B*07-epitope, and HLA-B*44-epitope complexes respectively. The RMSD plots of the reference complexes are studied as control and these complexes are also found to be stable throughout the simulation time as shown in Supplementary Fig. S1 online.

**Figure 7:**
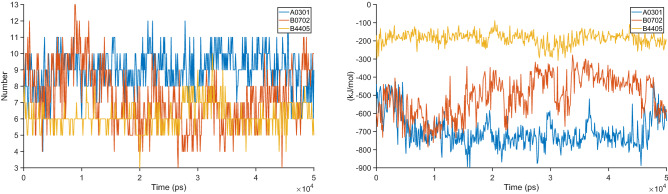
The interaction plots. Hydrogen bonds (left), short-range coulombic interactions (right).

Further, the short-range coulombic interaction and hydrogen bonding between the epitope and the HLA were analyzed to reveal the interaction stability of the epitope towards the corresponding HLA. The interaction plots as shown in Fig. 7 further confirm that the epitopes form a stable complex with strong bonding with the corresponding HLA.

While identifying the potential vaccine epitopes from SARS-CoV-2, this study considers all the epitopes that are not an identical subsequence to the human proteome as a foreign sequence. This could lead to the epitopes showing immune camouflaging that is generally seen in pathogens as an immune evasion strategy. This immune evasion is achieved by adopting amino acid configurations recognized by autologous regulatory T cells of the hosts which are known for their immune-modulating capabilities^20^. Apart from immune camouflaging, cross-reactivity of the T cells is also a major concern where the TCR recognizes more than one peptide-MHC complex. Although cross-reactivity is an essential feature of the immune system for biological robustness, in this case where only completely identical peptide sequences are filtered out to identify the epitope candidates, the autologous peptides could also be identified for an immune response especially in the case of an engineered TCR^21,22^.

The identified epitopes could be further subjected to in-vitro and in-vivo analysis after considering the limitations. Although epitopes play an important role in immune response, epitopes alone are not sufficient to develop vaccines as they cannot stimulate the immune system sufficiently. So, adjuvants such as biopolymers and nanoparticles are used besides an epitope in developing a peptide-based vaccine prototype for high immune response. These peptide-based vaccine prototypes should be further tested on a cell line for their physiological, biological, and chemical effects leading to cytotoxicity using in-vitro cytotoxicity assays such as in-vitro titration of live organisms, enzyme-linked immunosorbent assay (ELISA), and in-vitro antigen-quantification tests. The promising prototypes could be further subjected to in-vivo studies using animal testing techniques to identify the immunogenic prototype through immunization of laboratory animals and titration of immune sera to measure their antibody response. In-vivo serology analysis methods can be used to measure antibodies in blood samples employing techniques such as enzyme-linked immunosorbent assay (ELISA) and a multiplex assay that could analyze multiple antigens simultaneously before proceeding to further clinical phase studies^23^.

Over the last few months, there have been various studies on the identification of immunogenic epitopes from SARS-CoV-2 using computational techniques. But, these studies focus on a particular viral protein^24–26^, identifying epitopes from SARS-CoV which shows high sequence similarity along with sequence conservation to SARS-CoV-2^27^ or considering viral sequences from diverse geographical regions^28^. Unlike the other studies, our analysis primarily targets the Indian population where an infectious disease could escalate very rapidly. We have also analyzed the conserved nature of the identified epitopes along with their interaction stability towards the HLAs at the peptide-binding groove considering a known bound peptide as a reference. This could significantly increase the predictability in an in-vivo environment.

## Conclusion

Designing a vaccine is of top priority in the time of a pandemic. In this study, we attempted to identify potential CTL epitopes from the SARS-CoV-2 Indian isolate for the Indian population using a bioinformatics approach.

The list of CTL epitopes was predicted using the NetCTLPan server which considers HLA binding affinity, TAP transport efficiency, and C-terminal cleavage to identify the epitopes. Further, Immunogenicity scores were calculated using the IEDB immunogenicity tool to identify the potential vaccine epitope candidates. The epitopes with positive immunogenic scores are subjected to peptide matching against the human proteome to check their foreignness to the human body. These unique immunogenic epitopes were further docked with their respective HLA molecule to study their interactions with the HLA molecule. The docking studies revealed that all the studied epitopes bind at the peptide-binding site of the HLA confirming their epitope candidacy. The identified epitopes were checked for their sequence conservation in the viral isolates from India and the HLA-epitope complexes were subjected to molecular dynamics simulation studies to analyze their interaction stability. The epitopes were observed to be highly conserved and the interactions between the HLA and the epitopes were seen to be very stable, further confirming their potency in vaccine development. The epitopes identified in this study can be further subjected to in-vitro and in-vivo studies to design a vaccine against the dreadful SARS-CoV-2.

## Supplementary Information

Supplementary Information 1.

Supplementary Information 2.

Supplementary Information 3.

## Data Availability

All the datasets generated and/or analyzed during the current study are either available in the figshare repository https://doi.org/10.6084/m9.figshare.12106737 or are included in this published article (and its Supplementary Information files). GenBank ID of the genome: MT050493.1. PDB ID for the structure of HLA-A3: 6O9C. PDB ID for the structure of HLA-B7: 6AT5. PDB ID for the structure of HLA-B44: 3KPS.
