# Supplementary figures and images for: Immunoinformatic based identification of cytotoxic T lymphocyte epitopes from the Indian isolate of SARS-CoV-2
Source: Sci Rep. 2021 Feb 25;11:4516. doi: 10.1038/s41598-021-83949-9 (PMC7907102; doi:10.1038/s41598-021-83949-9)

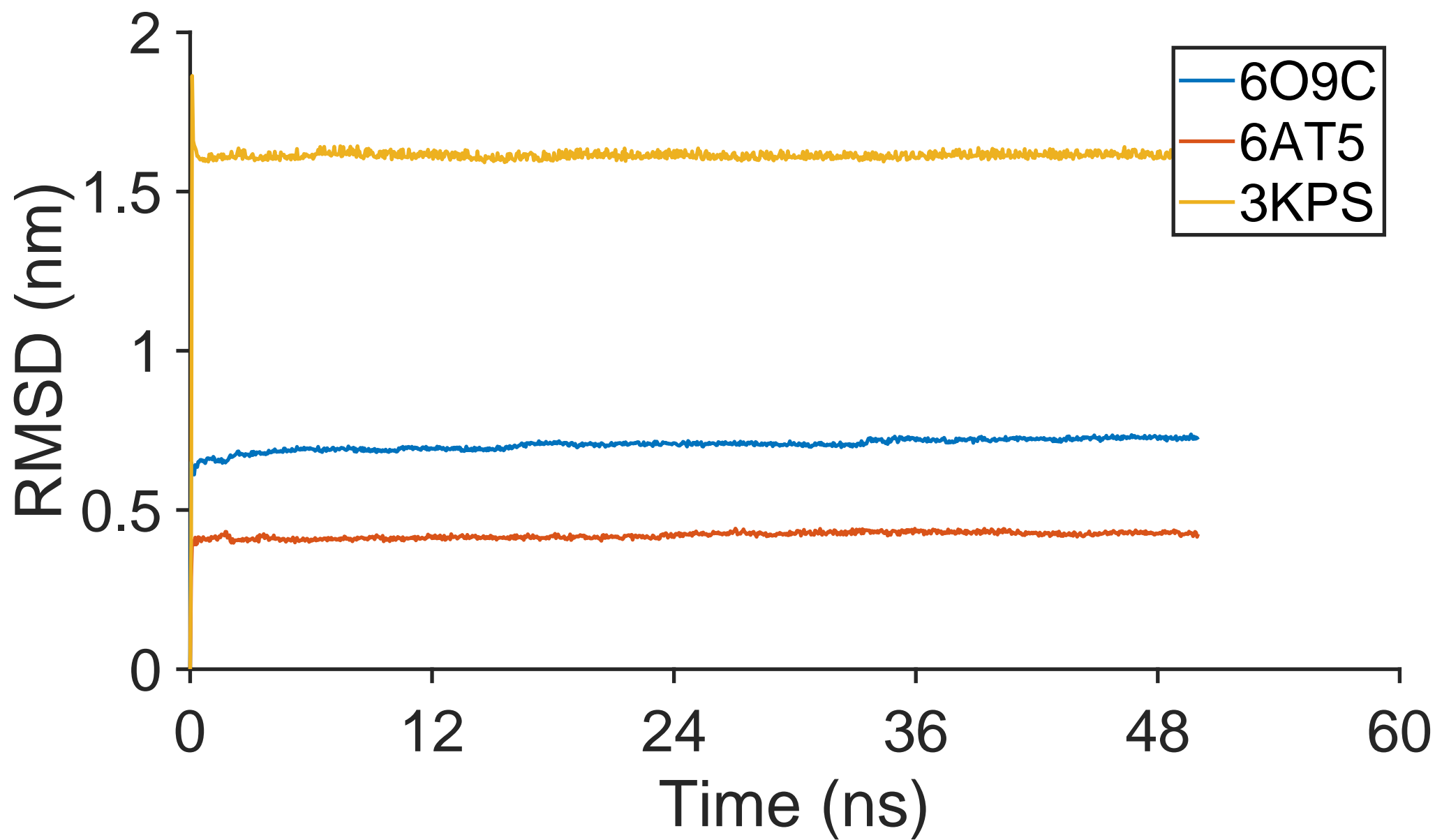

Fig. S1. The RMSD plot of the control HLA complexes

Supplement: Supplementary file 2 — Supplementary Information 2. [file 41598_2021_83949_MOESM2_ESM.pdf]
