# Supplementary material for: Immunoinformatic based identification of cytotoxic T lymphocyte epitopes from the Indian isolate of SARS-CoV-2
Source: Sci Rep. 2021 Feb 25;11:4516. doi: 10.1038/s41598-021-83949-9 (PMC7907102; doi:10.1038/s41598-021-83949-9)
Supplement: Supplementary file 3 — Supplementary Information 3. [file 41598_2021_83949_MOESM3_ESM.docx]

**Immunoinformatic based identification of cytotoxic T lymphocyte epitopes from the Indian isolate of SARS-CoV-2**

**Viswajit Mulpuru^1^, Nidhi Mishra^2,^***

^1^ Indian Institute of Information Technology Allahabad, Department of Applied Science, Prayagraj, 211012, India.

^2^ Indian Institute of Information Technology Allahabad, Department of Applied Science, Prayagraj, 211012, India.

*nidhimishra@iiita.ac.in

**Supplementary Table S1: The CTL epitopes as predicted by NetCTLpan**

Supplementary Table S1.a: Epitopes for HLA-A3

| **Protein** | **Epitope** | **Score** |
| --- | --- | --- |
| orf1ab polyprotein | VMYMGTLSY | 1.10186 |
| orf1ab polyprotein | RLYYDSMSY | 1.08196 |
| orf1ab polyprotein | KLFDRYFKY | 1.05478 |
| orf1ab polyprotein | STFNVPMEK | 1.00024 |
| orf1ab polyprotein | KLFAAETLK | 0.98899 |
| orf1ab polyprotein | SMMGFKMNY | 0.98124 |
| orf1ab polyprotein | HLYLQYIRK | 0.95284 |
| orf1ab polyprotein | GVYSVIYLY | 0.89655 |
| orf1ab polyprotein | HLMGWDYPK | 0.89529 |
| orf1ab polyprotein | RQFHQKLLK | 0.89498 |
| orf1ab polyprotein | ASMPTTIAK | 0.89119 |
| orf1ab polyprotein | VTNNTFTLK | 0.88863 |
| orf1ab polyprotein | ALAYYNTTK | 0.88487 |
| orf1ab polyprotein | VLSGHNLAK | 0.88202 |
| orf1ab polyprotein | QTFFKLVNK | 0.87836 |
| orf1ab polyprotein | KMQRMLLEK | 0.8742 |
| orf1ab polyprotein | TMLFTMLRK | 0.86696 |
| orf1ab polyprotein | MMSAPPAQY | 0.86352 |
| orf1ab polyprotein | KSAGFPFNK | 0.86012 |
| orf1ab polyprotein | ALCTFLLNK | 0.85256 |
| orf1ab polyprotein | QMCLSTLMK | 0.83986 |
| orf1ab polyprotein | LMNVLTLVY | 0.83735 |
| orf1ab polyprotein | AMRPNFTIK | 0.83685 |
| orf1ab polyprotein | MTNRQFHQK | 0.83439 |
| orf1ab polyprotein | GVAMPNLYK | 0.83099 |
| orf1ab polyprotein | LLMPLKAPK | 0.82852 |
| orf1ab polyprotein | YIFFASFYY | 0.8274 |
| orf1ab polyprotein | IINNTVYTK | 0.82687 |
| orf1ab polyprotein | QLYLGGMSY | 0.82596 |
| orf1ab polyprotein | LLNKEMYLK | 0.81902 |
| orf1ab polyprotein | VVYRGITTY | 0.81876 |
| orf1ab polyprotein | AIDAYPLTK | 0.8123 |
| orf1ab polyprotein | AIMQLFFSY | 0.80874 |
| orf1ab polyprotein | TSFGPLVRK | 0.80599 |
| orf1ab polyprotein | RLISMMGFK | 0.80566 |
| orf1ab polyprotein | KLMVVIPDY | 0.80491 |
| orf1ab polyprotein | ALRANSAVK | 0.80131 |
| orf1ab polyprotein | GLQPSVGPK | 0.80051 |
| orf1ab polyprotein | YLKSPNFSK | 0.80016 |
| orf1ab polyprotein | VLDMCASLK | 0.79643 |
| orf1ab polyprotein | ALKYLPIDK | 0.79419 |
| orf1ab polyprotein | MVMCGGSLY | 0.78854 |
| orf1ab polyprotein | RVVRSIFSR | 0.78842 |
| orf1ab polyprotein | TLKGGAPTK | 0.78727 |
| orf1ab polyprotein | RLRAKHYVY | 0.7823 |
| orf1ab polyprotein | SAFAMMFVK | 0.7788 |
| orf1ab polyprotein | SLREVRTIK | 0.77664 |
| orf1ab polyprotein | YMSALNHTK | 0.77504 |
| orf1ab polyprotein | AVLQSGFRK | 0.77304 |
| orf1ab polyprotein | SLVPGFNEK | 0.77198 |
| orf1ab polyprotein | VIYLYLTFY | 0.77021 |
| orf1ab polyprotein | TTIKPVTYK | 0.76767 |
| orf1ab polyprotein | VLHDIGNPK | 0.7675 |
| orf1ab polyprotein | GTFTCASEY | 0.7662 |
| orf1ab polyprotein | KTIQPRVEK | 0.76579 |
| orf1ab polyprotein | YSYATHSDK | 0.76521 |
| orf1ab polyprotein | TISLAGSYK | 0.76239 |
| orf1ab polyprotein | VLLRKNGNK | 0.7589 |
| orf1ab polyprotein | MSYYCKSHK | 0.75739 |
| orf1ab polyprotein | MLVYCFLGY | 0.75646 |
| orf1ab polyprotein | RVCTNYMPY | 0.75355 |
| orf1ab polyprotein | KLTDNVYIK | 0.75162 |
| orf1ab polyprotein | KVVKVTIDY | 0.74816 |
| orf1ab polyprotein | KNFKSVLYY | 0.73597 |
| orf1ab polyprotein | ALILAYCNK | 0.73362 |
| orf1ab polyprotein | RIDKVLNEK | 0.73328 |
| orf1ab polyprotein | FSYVGCHNK | 0.72967 |
| orf1ab polyprotein | MASLVLARK | 0.72867 |
| orf1ab polyprotein | TVKPGNFNK | 0.7286 |
| orf1ab polyprotein | MSLSEQLRK | 0.7285 |
| orf1ab polyprotein | AVAKHDFFK | 0.72709 |
| orf1ab polyprotein | ALGGSVAIK | 0.72496 |
| orf1ab polyprotein | QTMLFTMLR | 0.72303 |
| orf1ab polyprotein | KMNYQVNGY | 0.71826 |
| orf1ab polyprotein | AQCFKMFYK | 0.71732 |
| orf1ab polyprotein | LTAVVIPTK | 0.71593 |
| orf1ab polyprotein | KLVLSVNPY | 0.71273 |
| orf1ab polyprotein | YLALYNKYK | 0.70978 |
| orf1ab polyprotein | TLQCIMLVY | 0.70767 |
| orf1ab polyprotein | VLITEGSVK | 0.70421 |
| orf1ab polyprotein | FSSEIIGYK | 0.70365 |
| orf1ab polyprotein | ASHMYCSFY | 0.70292 |
| orf1ab polyprotein | TVAYFNMVY | 0.7028 |
| orf1ab polyprotein | LVAEWFLAY | 0.70209 |
| orf1ab polyprotein | TLSYEQFKK | 0.70031 |
| orf1ab polyprotein | ALCEKALKY | 0.6999 |
| orf1ab polyprotein | TVIEVQGYK | 0.69965 |
| orf1ab polyprotein | NIFGTVYEK | 0.69679 |
| orf1ab polyprotein | MSALNHTKK | 0.69616 |
| orf1ab polyprotein | GVTFQSAVK | 0.69494 |
| surface glycoprotein | RLFRKSNLK | 1.03511 |
| surface glycoprotein | GVYFASTEK | 0.93981 |
| surface glycoprotein | TLKSFTVEK | 0.89372 |
| surface glycoprotein | VTYVPAQEK | 0.83137 |
| surface glycoprotein | MTSCCSCLK | 0.79628 |
| surface glycoprotein | QIYKTPPIK | 0.78044 |
| surface glycoprotein | KVFRSSVLH | 0.76123 |
| surface glycoprotein | VLKGVKLHY | 0.7496 |
| surface glycoprotein | RISNCVADY | 0.73792 |
| surface glycoprotein | ASANLAATK | 0.7306 |
| surface glycoprotein | TLADAGFIK | 0.71153 |
| surface glycoprotein | GVYYHKNNK | 0.71101 |
| orf3a protein | IMRLWLCWK | 0.84874 |
| orf3a protein | HVTFFIYNK | 0.78979 |
| orf3a protein | SASKIITLK | 0.78541 |
| orf3a protein | HSYFTSDYY | 0.73943 |
| orf3a protein | ASKIITLKK | 0.7172 |
| orf3a protein | FLYLYALVY | 0.71025 |
| envelope protein | SFYVYSRVK | 0.70636 |
| membrane glycoprotein | ATSRTLSYY | 0.78018 |
| membrane glycoprotein | TSRTLSYYK | 0.72221 |
| orf6 protein | KVSIWNLDY | 0.74399 |
| orf7a protein | TLCFTLKRK | 0.6998 |
| orf8 protein | SLVVRCSFY | 0.70862 |
| nucleocapsid phosphoprotein | KTFPPTEPK | 0.94532 |

Supplementary Table S1.b: Epitopes for HLA-B7

| **Protein** | **Epitope** | **Score** |
| --- | --- | --- |
| orf1ab polyprotein | IPRRNVATL | 1.04681 |
| orf1ab polyprotein | IPVAYRKVL | 0.9884 |
| orf1ab polyprotein | NPAWRKAVF | 0.98075 |
| orf1ab polyprotein | KPVETSNSF | 0.97891 |
| orf1ab polyprotein | FPPTSFGPL | 0.96646 |
| orf1ab polyprotein | KPNELSRVL | 0.95603 |
| orf1ab polyprotein | VPHISRQRL | 0.91115 |
| orf1ab polyprotein | SPYNSQNAV | 0.90513 |
| orf1ab polyprotein | QPGQTFSVL | 0.89577 |
| orf1ab polyprotein | MPYFFTLLL | 0.88606 |
| orf1ab polyprotein | RPDTRYVLM | 0.84832 |
| orf1ab polyprotein | VPMEKLKTL | 0.84765 |
| orf1ab polyprotein | LPGCDGGSL | 0.83949 |
| orf1ab polyprotein | YLRKHFSMM | 0.8382 |
| orf1ab polyprotein | VPGLPGTIL | 0.82856 |
| orf1ab polyprotein | CPACHNSEV | 0.80434 |
| orf1ab polyprotein | NARDGCVPL | 0.80287 |
| orf1ab polyprotein | KPYIKWDLL | 0.80098 |
| orf1ab polyprotein | RPPLNRNYV | 0.78524 |
| orf1ab polyprotein | APLLSAGIF | 0.78492 |
| orf1ab polyprotein | HPTQAPTHL | 0.77628 |
| orf1ab polyprotein | MPASWVMRI | 0.76746 |
| orf1ab polyprotein | LPVNVAFEL | 0.7657 |
| orf1ab polyprotein | IPLTTAAKL | 0.76004 |
| orf1ab polyprotein | MPLKAPKEI | 0.73855 |
| orf1ab polyprotein | KPASRELKV | 0.73782 |
| orf1ab polyprotein | FPLKLRGTA | 0.72903 |
| orf1ab polyprotein | IPTITQMNL | 0.7271 |
| orf1ab polyprotein | IPKEEVKPF | 0.72182 |
| orf1ab polyprotein | AARVVRSIF | 0.71783 |
| orf1ab polyprotein | YPNASFDNF | 0.71011 |
| orf1ab polyprotein | APYIVGDVV | 0.70862 |
| orf1ab polyprotein | TPAFDKSAF | 0.70808 |
| orf1ab polyprotein | VPHVGEIPV | 0.70234 |
| orf1ab polyprotein | TPRDLGACI | 0.69421 |
| orf1ab polyprotein | FPFNKWGKA | 0.69224 |
| orf1ab polyprotein | APISAMVRM | 0.69003 |
| orf1ab polyprotein | KPVPEVKIL | 0.68934 |
| orf1ab polyprotein | YPKLQSSQA | 0.67982 |
| orf1ab polyprotein | YVFCTVNAL | 0.67375 |
| orf1ab polyprotein | YIRKLHDEL | 0.66979 |
| orf1ab polyprotein | QLRVIGHSM | 0.66829 |
| orf1ab polyprotein | TPHTVLQAV | 0.66691 |
| orf1ab polyprotein | LPSLATVAY | 0.66527 |
| orf1ab polyprotein | YVRNLQHRL | 0.66264 |
| surface glycoprotein | LPPAYTNSF | 0.93481 |
| surface glycoprotein | SPRRARSVA | 0.88471 |
| surface glycoprotein | MIAQYTSAL | 0.88465 |
| surface glycoprotein | IPTNFTISV | 0.76045 |
| surface glycoprotein | LPFNDGVYF | 0.72745 |
| surface glycoprotein | WPWYIWLGF | 0.68398 |
| orf3a protein | IPIQASLPF | 0.96367 |
| orf3a protein | APFLYLYAL | 0.91151 |
| membrane glycoprotein | RLFARTRSM | 0.80922 |
| membrane glycoprotein | HLRIAGHHL | 0.75909 |
| orf7a protein | RARSVSPKL | 0.7647 |
| orf8 protein | EPKLGSLVV | 0.67708 |
| nucleocapsid phosphoprotein | FPRGQGVPI | 1.05328 |
| nucleocapsid phosphoprotein | SPRWYFYYL | 1.00321 |
| nucleocapsid phosphoprotein | LPNNTASWF | 0.73406 |
| nucleocapsid phosphoprotein | KPRQKRTAT | 0.67 |
| orf10 protein | FPFTIYSLL | 0.80506 |

Supplementary Table S1.c: Epitopes for HLA-B44

| **Protein** | **Epitope** | **Score** |
| --- | --- | --- |
| orf1ab polyprotein | AEWFLAYIL | 1.05299 |
| orf1ab polyprotein | QEYADVFHL | 0.99723 |
| orf1ab polyprotein | RELHLSWEV | 0.98366 |
| orf1ab polyprotein | GEYSHVVAF | 0.98311 |
| orf1ab polyprotein | YELQTPFEI | 0.98281 |
| orf1ab polyprotein | HEVLLAPLL | 0.98237 |
| orf1ab polyprotein | CEFCGTENL | 0.97958 |
| orf1ab polyprotein | GEAANFCAL | 0.97538 |
| orf1ab polyprotein | TEVVGDIIL | 0.96605 |
| orf1ab polyprotein | FELEDFIPM | 0.95557 |
| orf1ab polyprotein | HEFCSQHTM | 0.94133 |
| orf1ab polyprotein | SEVGPEHSL | 0.93802 |
| orf1ab polyprotein | HEGKTFYVL | 0.93282 |
| orf1ab polyprotein | REVLSDREL | 0.92073 |
| orf1ab polyprotein | HEETIYNLL | 0.90666 |
| orf1ab polyprotein | AELAKNVSL | 0.90417 |
| orf1ab polyprotein | GEVITFDNL | 0.88458 |
| orf1ab polyprotein | GETLPTEVL | 0.87666 |
| orf1ab polyprotein | EETGLLMPL | 0.8759 |
| orf1ab polyprotein | NETLVTMPL | 0.87157 |
| orf1ab polyprotein | GEFKLASHM | 0.8409 |
| orf1ab polyprotein | AEYHNESGL | 0.82636 |
| orf1ab polyprotein | REHEHEIAW | 0.82399 |
| orf1ab polyprotein | REQIDGYVM | 0.79457 |
| orf1ab polyprotein | LEMELTPVV | 0.78537 |
| orf1ab polyprotein | KENSYTTTI | 0.78397 |
| orf1ab polyprotein | LENVAFNVV | 0.769 |
| orf1ab polyprotein | FEEAALCTF | 0.76465 |
| orf1ab polyprotein | AESHVDTDL | 0.76428 |
| orf1ab polyprotein | SEFDRDAAM | 0.75706 |
| orf1ab polyprotein | VEKGVLPQL | 0.75496 |
| orf1ab polyprotein | LEQYVFCTV | 0.75398 |
| orf1ab polyprotein | NELSRVLGL | 0.74691 |
| orf1ab polyprotein | TEEVGHTDL | 0.74426 |
| orf1ab polyprotein | TEHSWNADL | 0.74138 |
| orf1ab polyprotein | VESCGNFKV | 0.74112 |
| orf1ab polyprotein | FENKTTLPV | 0.74075 |
| orf1ab polyprotein | YENFNQHEV | 0.74035 |
| orf1ab polyprotein | NESGLKTIL | 0.737 |
| orf1ab polyprotein | WEPEFYEAM | 0.73663 |
| orf1ab polyprotein | LEQPTSEAV | 0.72918 |
| orf1ab polyprotein | SELLTPLGI | 0.71934 |
| orf1ab polyprotein | VEYCPIFFI | 0.71615 |
| orf1ab polyprotein | TQWSLFFFL | 0.71219 |
| orf1ab polyprotein | FEHIVYGDF | 0.70234 |
| orf1ab polyprotein | REVRTIKVF | 0.69523 |
| orf1ab polyprotein | SEIIGYKAI | 0.68964 |
| orf1ab polyprotein | IERFVSLAI | 0.68494 |
| orf1ab polyprotein | VEVQPQLEM | 0.68333 |
| orf1ab polyprotein | CEIVGGQIV | 0.67243 |
| orf1ab polyprotein | LEFGATSAA | 0.65558 |
| orf1ab polyprotein | QEILGTVSW | 0.65318 |
| orf1ab polyprotein | RQWLPTGTL | 0.65308 |
| orf1ab polyprotein | LEPEYFNSV | 0.63942 |
| orf1ab polyprotein | IEVQGYKSV | 0.63372 |
| orf1ab polyprotein | SEFSSLPSY | 0.63075 |
| orf1ab polyprotein | DEVARDLSL | 0.62591 |
| orf1ab polyprotein | SQLGGLHLL | 0.625 |
| orf1ab polyprotein | TEVLTEEVV | 0.62051 |
| orf1ab polyprotein | TEVPANSTV | 0.61847 |
| orf1ab polyprotein | VETSNSFDV | 0.61627 |
| orf1ab polyprotein | EEAALCTFL | 0.61414 |
| orf1ab polyprotein | LEGNFYGPF | 0.61298 |
| orf1ab polyprotein | FELWAKRNI | 0.60584 |
| orf1ab polyprotein | FEIKLAKKF | 0.60515 |
| surface glycoprotein | AEIRASANL | 0.9586 |
| surface glycoprotein | FEYVSQPFL | 0.91659 |
| surface glycoprotein | KEIDRLNEV | 0.70996 |
| surface glycoprotein | FERDISTEI | 0.7009 |
| surface glycoprotein | GEVFNATRF | 0.66471 |
| surface glycoprotein | IEDLLFNKV | 0.63531 |
| surface glycoprotein | AEVQIDRLI | 0.61415 |
| surface glycoprotein | YEQYIKWPW | 0.60738 |
| envelope protein | SEETGTLIV | 0.71532 |
| membrane glycoprotein | SELVIGAVI | 0.77016 |
| orf7a protein | QELYSPIFL | 0.72089 |

**Supplementary Table S2: The immunogenicity score as predicted by (IEDB) immunogenicity tool**

| Epitope | Immunogenicity Score |
| --- | --- |
| LVAEWFLAY | 0.45285 |
| WPWYIWLGF | 0.41673 |
| REHEHEIAW | 0.37218 |
| HVTFFIYNK | 0.36278 |
| SPRWYFYYL | 0.34101 |
| FELEDFIPM | 0.33479 |
| FSSEIIGYK | 0.32837 |
| SLREVRTIK | 0.31699 |
| WEPEFYEAM | 0.31503 |
| GEVITFDNL | 0.31026 |
| TEVVGDIIL | 0.2989 |
| IMRLWLCWK | 0.29482 |
| KVSIWNLDY | 0.29343 |
| AEWFLAYIL | 0.29037 |
| VPHVGEIPV | 0.28513 |
| TLADAGFIK | 0.28158 |
| HLRIAGHHL | 0.26692 |
| VVYRGITTY | 0.2646 |
| SELVIGAVI | 0.25658 |
| MPYFFTLLL | 0.2513 |
| APYIVGDVV | 0.24766 |
| KSAGFPFNK | 0.24538 |
| TEVLTEEVV | 0.24291 |
| LPVNVAFEL | 0.24122 |
| KLFAAETLK | 0.236 |
| LTAVVIPTK | 0.23304 |
| VEYCPIFFI | 0.22623 |
| GEVFNATRF | 0.22473 |
| FEHIVYGDF | 0.2227 |
| AMRPNFTIK | 0.22006 |
| QEYADVFHL | 0.21633 |
| SEETGTLIV | 0.2095 |
| LENVAFNVV | 0.2064 |
| NIFGTVYEK | 0.20414 |
| HLMGWDYPK | 0.20408 |
| SLVPGFNEK | 0.19848 |
| SEFDRDAAM | 0.18748 |
| HEETIYNLL | 0.18574 |
| TEEVGHTDL | 0.18321 |
| VLHDIGNPK | 0.18238 |
| IPTNFTISV | 0.17229 |
| TQWSLFFFL | 0.17203 |
| REQIDGYVM | 0.17082 |
| VTNNTFTLK | 0.16567 |
| KEIDRLNEV | 0.15852 |
| IPRRNVATL | 0.15714 |
| KLMVVIPDY | 0.15362 |
| QLRVIGHSM | 0.15048 |
| KPYIKWDLL | 0.14347 |
| KLTDNVYIK | 0.14212 |
| ASMPTTIAK | 0.14136 |
| NPAWRKAVF | 0.14013 |
| RQWLPTGTL | 0.13776 |
| LEGNFYGPF | 0.13713 |
| GETLPTEVL | 0.1358 |
| YIFFASFYY | 0.13481 |
| CEFCGTENL | 0.13401 |
| GEAANFCAL | 0.13333 |
| VPGLPGTIL | 0.13146 |
| KTFPPTEPK | 0.1306 |
| CEIVGGQIV | 0.12964 |
| RPDTRYVLM | 0.12154 |
| LPFNDGVYF | 0.11767 |
| TSFGPLVRK | 0.11594 |
| ALCTFLLNK | 0.11198 |
| RLFARTRSM | 0.11133 |
| RELHLSWEV | 0.10786 |
| FERDISTEI | 0.10425 |
| LEPEYFNSV | 0.10163 |
| TVIEVQGYK | 0.10155 |
| TEHSWNADL | 0.09962 |
| VLITEGSVK | 0.09616 |
| ASKIITLKK | 0.0947 |
| FELWAKRNI | 0.09429 |
| EEAALCTFL | 0.09168 |
| GVYFASTEK | 0.09023 |
| ASANLAATK | 0.08792 |
| IINNTVYTK | 0.08761 |
| LEQYVFCTV | 0.08626 |
| YENFNQHEV | 0.08616 |
| AESHVDTDL | 0.08565 |
| AARVVRSIF | 0.0854 |
| AEVQIDRLI | 0.08452 |
| LEMELTPVV | 0.08425 |
| KLFDRYFKY | 0.08004 |
| LMNVLTLVY | 0.07994 |
| MLVYCFLGY | 0.07782 |
| YVFCTVNAL | 0.07781 |
| FEEAALCTF | 0.07671 |
| VIYLYLTFY | 0.07476 |
| SQLGGLHLL | 0.07388 |
| REVRTIKVF | 0.07068 |
| LPSLATVAY | 0.06748 |
| YEQYIKWPW | 0.06574 |
| YELQTPFEI | 0.0645 |
| TPRDLGACI | 0.06174 |
| SEVGPEHSL | 0.06159 |
| KTIQPRVEK | 0.0579 |
| FPFTIYSLL | 0.05708 |
| LPNNTASWF | 0.05582 |
| ALAYYNTTK | 0.05473 |
| NARDGCVPL | 0.04973 |
| SEIIGYKAI | 0.0475 |
| TLKGGAPTK | 0.04725 |
| FSYVGCHNK | 0.04611 |
| AIDAYPLTK | 0.04585 |
| TVKPGNFNK | 0.04077 |
| SPRRARSVA | 0.0402 |
| QEILGTVSW | 0.03976 |
| FLYLYALVY | 0.03563 |
| KPVPEVKIL | 0.03436 |
| ALGGSVAIK | 0.03364 |
| IERFVSLAI | 0.03257 |
| APFLYLYAL | 0.03254 |
| RVVRSIFSR | 0.0318 |
| ALKYLPIDK | 0.03032 |
| LEFGATSAA | 0.02998 |
| MASLVLARK | 0.02816 |
| VTYVPAQEK | 0.02711 |
| SELLTPLGI | 0.02304 |
| RPPLNRNYV | 0.02004 |
| VLSGHNLAK | 0.01931 |
| YMSALNHTK | 0.01876 |
| DEVARDLSL | 0.01803 |
| ALILAYCNK | 0.01738 |
| IPLTTAAKL | 0.01711 |
| AVAKHDFFK | 0.01528 |
| HSYFTSDYY | 0.01523 |
| HLYLQYIRK | 0.01392 |
| HEVLLAPLL | 0.01243 |
| SASKIITLK | 0.01046 |
| TMLFTMLRK | 0.00758 |
| TLKSFTVEK | 0.00741 |
| MTNRQFHQK | 0.007 |
| AEIRASANL | 0.00689 |
| FPPTSFGPL | 0.00668 |
| TPHTVLQAV | 0.00442 |
| QELYSPIFL | 0.00186 |
| YPNASFDNF | 0.00131 |
| KPNELSRVL | -8.00E-05 |
| AEYHNESGL | -0.00052 |
| KVVKVTIDY | -0.00158 |
| FPRGQGVPI | -0.00164 |
| TVAYFNMVY | -0.00719 |
| GVYSVIYLY | -0.01179 |
| SLVVRCSFY | -0.01663 |
| MPASWVMRI | -0.01717 |
| GTFTCASEY | -0.01973 |
| YSYATHSDK | -0.02024 |
| IPKEEVKPF | -0.02137 |
| TLCFTLKRK | -0.0241 |
| HPTQAPTHL | -0.02464 |
| RISNCVADY | -0.02787 |
| STFNVPMEK | -0.02845 |
| APLLSAGIF | -0.03267 |
| LPPAYTNSF | -0.03341 |
| QTFFKLVNK | -0.03358 |
| VPHISRQRL | -0.03548 |
| REVLSDREL | -0.0358 |
| IEDLLFNKV | -0.03602 |
| HEGKTFYVL | -0.037 |
| GEYSHVVAF | -0.03961 |
| ALRANSAVK | -0.04872 |
| SFYVYSRVK | -0.05119 |
| YVRNLQHRL | -0.05201 |
| LPGCDGGSL | -0.05781 |
| LEQPTSEAV | -0.05933 |
| IPVAYRKVL | -0.05999 |
| KMNYQVNGY | -0.06542 |
| FPFNKWGKA | -0.0674 |
| MSALNHTKK | -0.06755 |
| MMSAPPAQY | -0.07023 |
| NELSRVLGL | -0.07037 |
| AIMQLFFSY | -0.07202 |
| QTMLFTMLR | -0.0723 |
| QPGQTFSVL | -0.07306 |
| NETLVTMPL | -0.0765 |
| IPTITQMNL | -0.0767 |
| KENSYTTTI | -0.08019 |
| GVTFQSAVK | -0.08099 |
| TEVPANSTV | -0.08269 |
| VEKGVLPQL | -0.08318 |
| TLSYEQFKK | -0.09616 |
| CPACHNSEV | -0.09726 |
| RLRAKHYVY | -0.10238 |
| YIRKLHDEL | -0.10333 |
| KPVETSNSF | -0.1059 |
| VESCGNFKV | -0.10824 |
| TLQCIMLVY | -0.11279 |
| TTIKPVTYK | -0.11514 |
| TSRTLSYYK | -0.11595 |
| ATSRTLSYY | -0.11604 |
| KMQRMLLEK | -0.11782 |
| AVLQSGFRK | -0.12032 |
| QIYKTPPIK | -0.12244 |
| NESGLKTIL | -0.12288 |
| RIDKVLNEK | -0.127 |
| EETGLLMPL | -0.12922 |
| FPLKLRGTA | -0.1314 |
| KLVLSVNPY | -0.13194 |
| SAFAMMFVK | -0.13601 |
| TISLAGSYK | -0.13664 |
| VLLRKNGNK | -0.14279 |
| RVCTNYMPY | -0.1429 |
| KPASRELKV | -0.14448 |
| GLQPSVGPK | -0.14888 |
| FENKTTLPV | -0.1606 |
| MSLSEQLRK | -0.16073 |
| TPAFDKSAF | -0.16766 |
| FEYVSQPFL | -0.17076 |
| VEVQPQLEM | -0.17386 |
| AQCFKMFYK | -0.17836 |
| APISAMVRM | -0.18539 |
| GVYYHKNNK | -0.18566 |
| MIAQYTSAL | -0.18768 |
| EPKLGSLVV | -0.18913 |
| VLKGVKLHY | -0.18916 |
| YLALYNKYK | -0.19231 |
| QLYLGGMSY | -0.19232 |
| GVAMPNLYK | -0.19241 |
| MVMCGGSLY | -0.19245 |
| KVFRSSVLH | -0.19839 |
| VETSNSFDV | -0.20414 |
| KPRQKRTAT | -0.20542 |
| IPIQASLPF | -0.20683 |
| VMYMGTLSY | -0.21438 |
| ALCEKALKY | -0.22528 |
| HEFCSQHTM | -0.23641 |
| AELAKNVSL | -0.24214 |
| YLKSPNFSK | -0.25122 |
| LLMPLKAPK | -0.25542 |
| SPYNSQNAV | -0.26045 |
| RQFHQKLLK | -0.26109 |
| SMMGFKMNY | -0.26388 |
| QMCLSTLMK | -0.26518 |
| GEFKLASHM | -0.27369 |
| RLFRKSNLK | -0.28759 |
| ASHMYCSFY | -0.29177 |
| LLNKEMYLK | -0.2965 |
| YLRKHFSMM | -0.30072 |
| KNFKSVLYY | -0.31276 |
| MPLKAPKEI | -0.31644 |
| VLDMCASLK | -0.33127 |
| VPMEKLKTL | -0.33601 |
| IEVQGYKSV | -0.3523 |
| RLISMMGFK | -0.36257 |
| MTSCCSCLK | -0.36816 |
| MSYYCKSHK | -0.38114 |
| RLYYDSMSY | -0.38391 |
| RARSVSPKL | -0.40056 |
| SEFSSLPSY | -0.40603 |
| FEIKLAKKF | -0.45577 |
| YPKLQSSQA | -0.55699 |
